# Exosome isolation and characterization for advanced diagnostic and therapeutic applications

**DOI:** 10.1016/j.mtbio.2025.101613

**Published:** 2025-02-25

**Authors:** Nobendu Mukerjee, Arghya Bhattacharya, Swastika Maitra, Mandeep Kaur, Subbulakshmi Ganesan, Shivang Mishra, Ayash Ashraf, Muhammad Rizwan, Kavindra Kumar Kesari, Tanveer A. Tabish, Nanasaheb D. Thorat

**Affiliations:** a Center for Global Health Research, Saveetha Medical College and Hospital, Saveetha Institute of Medical and Technical Sciences, Chennai, India; b Department of Pharmacology, Bengal School of Technology, West Bengal, Kolkata, 712102, India; c Department of Sciences, Vivekananda Global University, Jaipur, Rajasthan, 303012, India; d Department of Chemistry and Biochemistry, School of Sciences, JAIN (Deemed to be University), Bangalore, Karnataka, India; e NIMS Institute of Pharmacy, NIMS University Rajasthan, Jaipur, India; f Chandigarh Pharmacy College, Chandigarh Group of College, Jhanjeri, Mohali, 140307, Punjab, India; g Department of Biomedical Engineering, Department of Ophthalmology, The University of Texas Southwestern Medical Center, Dallas, TX, USA; h Department of Applied Physics, Aalto University, Finland; i Radcliffe Department of Medicine, University of Oxford, OX3 7BN, United Kingdom; j Department of Physics and Bernal Institute, University of Limerick, Castletroy, Limerick V94T9PX, Ireland; k Limerick Digital Cancer Research Centre (LDCRC) University of Limerick, Castletroy, Limerick, V94T9PX, Ireland; l Centre for Infectious Diseases & Microbiology, School of Public Health Sciences and Technology, Malla Reddy Vishwavidyapeeth, Hyderabad 500 055, Telangana, India

**Keywords:** Exosome isolation, Ultracentrifugation, Microfluidics, Immunoaffinity capture, Exosome therapeutics

## Abstract

Advancements in exosome isolation technologies are pivotal for transforming personalized medicine and enhancing clinical diagnostics. Exosomes, small extracellular vesicles with diameters ranging between 30 and 150 nm, are secreted into bodily fluids by a variety of cells and play essential roles in intercellular communication. These vesicles facilitate the transfer of nucleic acids, lipids, and proteins, affecting a wide range of biological and pathological processes. Given their importance in disease diagnostics, therapy, and as biomarkers, there has been a surge in developing methods to isolate them from fluids such as urine, saliva, blood, and cerebrospinal fluid. While traditional isolation techniques like ultracentrifugation and polymer-based precipitation have been foundational, recent technological advances have introduced more precise methods like microfluidics and immunoaffinity capture. These newer methods enable high-throughput and specific exosome isolation by targeting surface markers, thus enhancing purity. However, challenges such as balancing purity with yield and the lack of standardized protocols across different laboratories persist, impacting the consistency of findings. By integrating advanced isolation techniques and discussing their implications in diagnostics and therapy, this review aims to catalyze further research and adoption of exosome-based technologies in medicine, marking a significant stride towards tailored healthcare solutions.

## Graphical abstract

**Figure:**
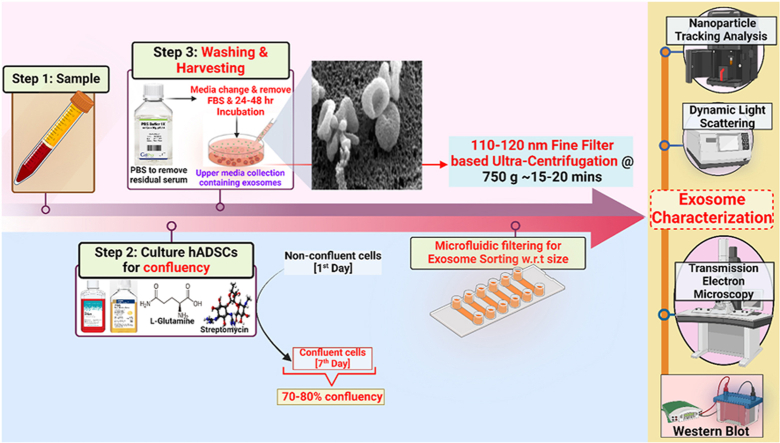

## 1 Introduction

Exosomes are minuscule extracellular vesicles that originate from the inward budding of endosomal compartments within cells, playing a pivotal role in intercellular communication. These vesicles, which range in size from 30 to 150 nm, encapsulate an array of biological molecules, including proteins, lipids, messenger RNA (mRNA), and microRNA (miRNA), reflective of their cell of origin [1,2]. The ability of exosomes to transport these molecules between cells positions them as crucial players in numerous physiological and pathological processes. In the realm of cancer theragnostic—a field combining therapy and diagnostics—exosomes have emerged as key elements due to their potential to serve as biomarkers and therapeutic delivery vehicles [3,4].

**Fig. 1:**
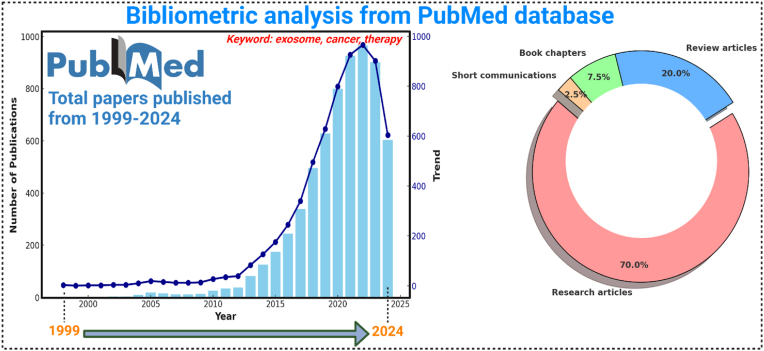
Bibliometric analysis focusing on the keywords “exosome, cancer, therapy” indicates a significant upward trend in the application of optical biosensors over the past twenty years from PubMed database.

Fig. 1 provides a bibliometric overview of publications retrieved from the PubMed database concerning the keywords “exosome,” “cancer,” and “therapy” spanning from 1999 to 2024. The data illustrates an upward trajectory in research interest, peaking in 2021, then slightly tapering off. The bar chart depicts annual publication volumes, reflecting an intensified focus on this research domain over the past decade. Additionally, the pie chart breaks down the publication types, showing that research articles dominate the field with 70 % of the total, followed by review articles at 20 %, book chapters at 7.5 %, and short communications at 2.5 %. This composition highlights a robust engagement with primary research, underlining the significance and ongoing developments in the application of exosomes in cancer therapy.

The isolation of exosomes from various body fluids such as blood, urine, saliva, cerebrospinal fluid, and breast milk is essential for leveraging their diagnostic and therapeutic potential. Each type of body fluid offers a unique perspective on the physiological and pathological state of the body, with exosomes acting as direct messengers carrying information about the health of their originating cells [5,6]. Various isolation techniques have been developed to extract these vesicles effectively, each tailored to address the specific challenges posed by different fluids. Techniques such as ultracentrifugation, size-based filtration, immunoaffinity capture, polymer precipitation, and advanced microfluidic technologies are employed to ensure the purity and integrity of isolated exosomes, making them suitable for clinical analysis [7,8].

In cancer theragnostic, exosomes are invaluable for both detecting malignancies and facilitating treatment. They carry genetic material and proteins from their cell of origin, including tumor-specific antigens and signaling molecules that can reveal the presence of cancer and provide insights into its progression and response to therapy [9]. Exosomes isolated from the blood of cancer patients can contain DNA, RNA, and proteins that mirror the genetic alterations and protein expressions of the tumor, offering a non-invasive means to monitor tumor dynamics and treatment efficacy over time [10,11]. Exosomes, small extracellular vesicles, play a vital role in cancer metastasis by facilitating intercellular communication, promoting tumor cell invasiveness, and preparing premetastatic niches. Their potential as diagnostic biomarkers and therapeutic targets offers promising avenues for cancer research and treatment [8,10].

**Fig. 2:**
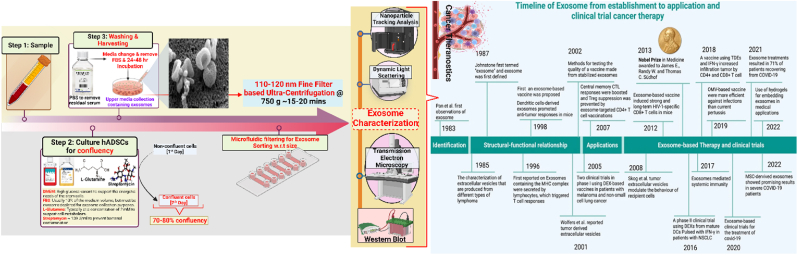
**Isolation, Characterization, and Cancer Theragnostic of Exosomes.** (Adapted with authorization under the Creative Commons CC BY 4.0 license, based on reference [15], Copyright © 2020 by the authors); **Timeline of Exosome based cancer therapy** (Adapted with authorization under the Creative Commons CC BY 4.0 license, based on reference [16], Copyright © 2020 by the authors).

Furthermore, the intrinsic ability of exosomes to naturally target specific tissues is being harnessed to develop targeted drug delivery systems. By engineering exosomes to carry therapeutic agents, such as chemotherapeutic drugs, RNA interference strategies, or CRISPR-Cas9 gene editing tools [12,13], researchers aim to reduce systemic toxicity and improve treatment outcomes. This approach holds promise for delivering therapies directly to tumor sites with high precision, minimizing adverse effects and enhancing the effectiveness of anticancer treatments [14] (Fig. 2).

The non-invasive nature of exosome collection from bodily fluids also presents an advantage in cancer treatment, where continuous monitoring of the disease state without repeated biopsies is desirable. This aspect is particularly beneficial for assessing tumor evolution, resistance to therapy, and for tailoring personalized treatment plans based on real-time data derived from exosomal contents [17,18]. The study of exosome isolation techniques and their applications in cancer theragnostic is rapidly advancing, offering exciting opportunities for enhancing diagnostic precision and therapeutic efficacy. These developments are poised to revolutionize the management of cancer, providing deeper insights into tumor biology and enabling more effective and personalized treatment strategies. The continuous evolution of exosome research underscores its potential to significantly impact the field of oncology, promising a future where cancer management is more dynamic, precise, and patient-friendly [[19], [20], [21]]. Previous reviews, for example, by Moshrefiravasjani et al. and Omrani et al. [7], have broadly described the critical functions of exosomes in cancer evolution, angiogenesis, immune suppression, and cellular signalling pathways, while our review focuses on advanced exosome isolation systems and their evolving diagnostic and therapeutic potentials. We highlight the functions of engineered exosomes in next generation therapeutic applications, innovative isolation approaches such as magnetic nanoparticle-based methods and particle-based flow cytometry, as well as the diagnostic potential of exosomes derived from liquid biopsies, for example cerebrospinal fluid, saliva, and breast milk. Such features, which are not discussed in earlier reviews, emphasize the key role of exosomes in both early disease diagnostics and targeted therapeutics.

This review introduces novel perspectives on exosome isolation techniques, focusing on advanced methods such as microfluidics and nanotechnology, which enhance isolation specificity and efficiency. It critically evaluates these technologies, identifies existing challenges, and suggests practical solutions, setting it apart from previous reviews. Additionally, this review discusses the implications of these advancements for clinical applications, emphasizing their potential in personalized medicine and targeted therapies. This innovative approach not only summarizes the current state but also guides future research and clinical practices in the rapidly evolving field of exosome research.

## 2 Exosome biogenesis

Exosome biogenesis is an intricate and highly regulated cellular process integral to the production of exosomes, which are small extracellular vesicles involved in various biological functions including intercellular communication. This process initiates within the endosomal system of a cell, starting with the formation of early endosomes through the inward budding of the plasma membrane. These early endosomes capture extracellular material along with membrane proteins, setting the stage for their subsequent transformation [[22], [23], [24]].

As these early endosomes mature into late endosomes, they undergo further morphological changes. Key among these changes is the inward budding of the late endosomal membrane, a process that results in the formation of multivesicular bodies (MVBs) [25]. The small vesicles that bud inward within the MVBs are known as intraluminal vesicles (ILVs), and these are the structures that eventually become exosomes. The content of ILVs is selectively enriched with a variety of biomolecules, such as signaling proteins, enzyme complexes, and nucleic acids. This selective packaging ensures that exosomes can effectively mediate specific signaling pathways once released from the cell [26].

The sorting of cargo into ILVs is a critical step in exosome biogenesis and involves multiple mechanisms. One prominent pathway is mediated by the Endosomal Sorting Complex Required for Transport (ESCRT) machinery. This machinery consists of several protein complexes (ESCRT-0, -I, -II, and -III) that successively to seize ubiquitinated proteins into the forming ILVs. Another significant pathway involves the lipid ceramide, which facilitates ILV formation through its role in inducing membrane curvature [27]. Additionally, tetraspanins, a family of proteins, contribute to cargo selection and membrane organization during exosome formation. Once the MVBs are laden with ILVs, they face a binary fate: they can either fuse with lysosomes, where their contents are degraded, or they can migrate to the plasma membrane [28]. At the plasma membrane, MVBs fuse and release their ILVs into the extracellular space as exosomes. This release mechanism is tightly controlled by cellular signaling pathways that dictate the timing and extent of exosome secretion based on the cell's physiological or pathological state [29].

**Fig. 3:**
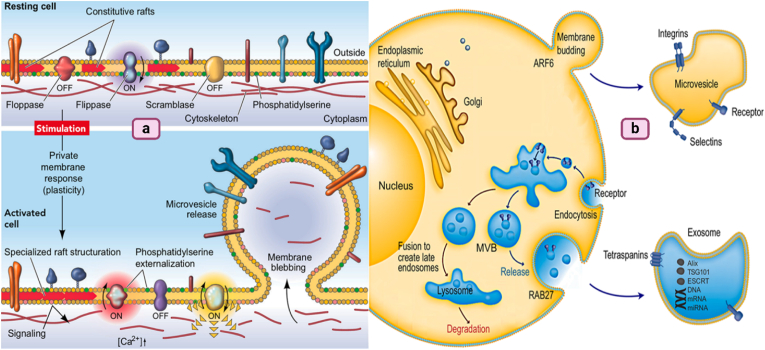
**(a) The formation of microvesicles.** (Adapted with authorization from Ref. [30], Copyright 2005 by the International Union of Physiological Sciences/The American Physiological Society); **(b) Cellular routes involved in the biogenesis and release of extracellular vehicles (EVs)**. (Adapted with authorization from Ref. [31], Copyright 2011 abcam.).

**Fig. 4:**
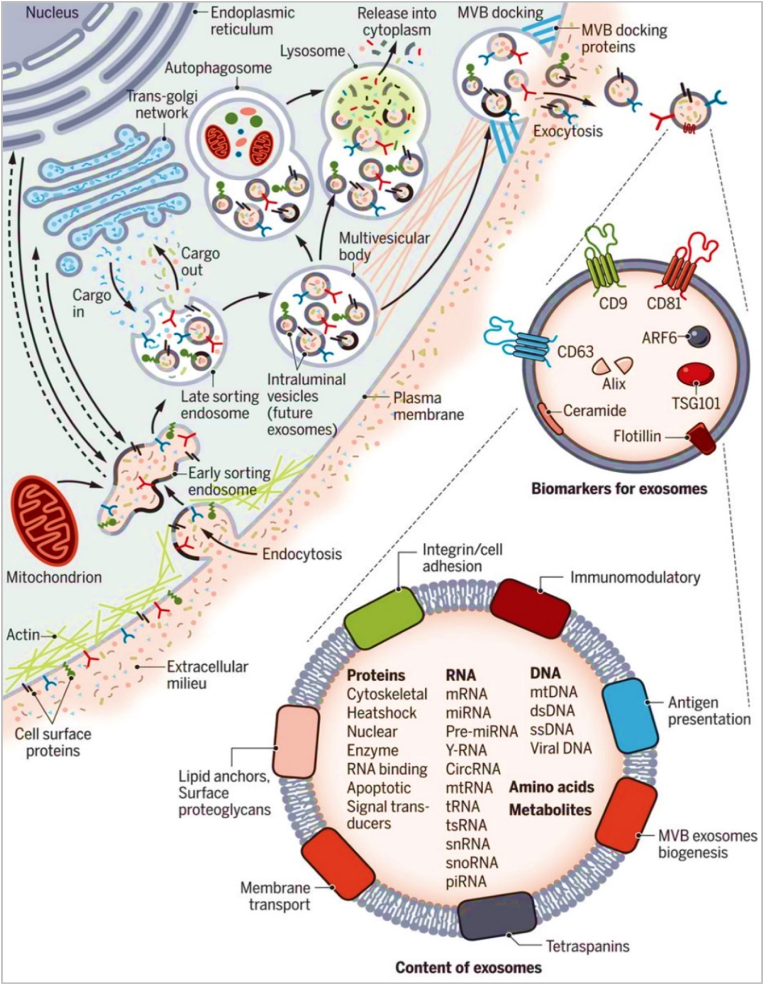
**Exosomes biogenesis pathway.** (Reproduced with permission under Creative Commons CC BY 4.0 license from Ref. [39] Copyright @ 2020 The Authors).

Phospholipids are not evenly spread across the plasma membrane's two layers. Three main proteins help maintain this uneven distribution: flippase, floppase, and lipid scramblase. Flippase moves certain phospholipids inward, while scramblase allows lipids to move in both directions, which helps mix them up. When a cell is activated, these lipids can shift around, leading to the release of tiny particles known as microvesicles [30]. Exosomes, another type of extracellular vesicle, are formed differently. They start as part of the plasma membrane that buds inward to form an early endosome, which then develops into a structure called an MVB (multivesicular body). Inside the MVB, more budding occurs, creating exosomes. These exosomes and microvesicles help cells communicate by carrying messages from one cell to another [31] (Fig. 3). Upon release, exosomes can influence a multitude of biological processes, from modulating immune responses and facilitating tumor progression to participating in the transfer of pathogenic agents [[32], [33], [34]]. This broad impact underscores the importance of understanding exosome biogenesis for both fundamental biology and medical applications. By exploring ways to manipulate exosome production or alter their molecular cargo, researchers hope to develop novel therapeutic strategies that exploit these natural vesicles to target diseases more effectively, offering a promising frontier in medical science [[35], [36], [37], [38]] (see Fig. 4).

## 3 Composition and biological significance of exosome

**Fig. 5:**
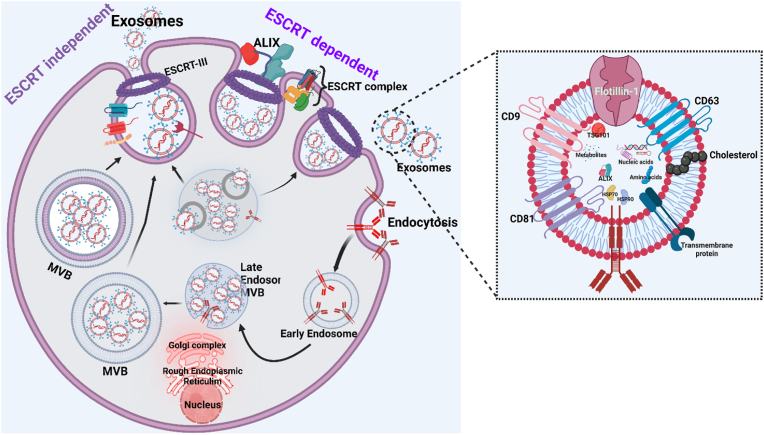
**Composition of Exosomes.** (Reproduced with permission under Creative Commons CC BY 4.0 license from Ref. [51] Copyright @ 2023 The Authors).

Exosomes are composed of a lipid bilayer that includes cholesterol, sphingolipids, ceramide, and phospholipids, encapsulating a variety of proteins such as tetraspanins, heat shock proteins, and signaling molecules, along with nucleic acids like mRNA fragments and microRNAs (Fig. 5). This complex molecular assembly allows exosomes to facilitate critical biological functions, including intercellular communication by transferring their cargo to recipient cells, thereby influencing gene expression and cellular behavior [[40], [41], [42], [43], [44]]. They play crucial roles in immune regulation, potentially activating or suppressing immune responses, aiding in tissue repair and regeneration by delivering growth factors, and even contributing to disease progression and metastasis in cancer by transporting oncogenic materials. Additionally, pathogens can exploit exosomes to spread infectious components across cells, underscoring their dual role in health and disease dynamics [[45], [46], [47], [48], [49], [50]].

Exosomes have a profound biological significance due to their role in mediating complex intercellular communications, which are essential for maintaining cellular and systemic homeostasis. These extracellular vesicles facilitate the transfer of molecular cargoes, such as proteins, lipids, and nucleic acids, directly influencing the physiological state and behavior of recipient cells [[52], [53], [54]]. This process is crucial in a variety of bodily functions, including immune responses where exosomes can modulate immune cell activation and suppress or promote inflammation, depending on their context and origin [55]. Moreover, exosomes contribute significantly to the progression and pathology of diseases. In cancer, for instance, tumor-derived exosomes can promote tumor growth and metastasis by manipulating the tumor microenvironment, enhancing tumor invasiveness, and helping cancer cells evade the immune system. In the context of neurodegenerative diseases, exosomes can spread pathogenic proteins like amyloid-beta or tau from diseased to healthy cells, potentially contributing to the progression of diseases such as cancer, Alzheimer's and Parkinson's [[56], [57], [58]]. Their role in such critical biological processes makes exosomes key targets for therapeutic interventions. Scientists are exploring how engineered exosomes can be used to deliver therapeutic agents selectively to specific cell types, offering a promising avenue for treatments with potentially fewer side effects compared to conventional therapies [[59], [60], [61], [62]]. Additionally, due to their stability in body fluids and the specificity of their molecular contents, exosomes are ideal candidates for biomarkers in liquid biopsy approaches, providing a non-invasive means to diagnose and monitor diseases, thus opening new frontiers in personalized medicine [[62], [63], [64], [65]].

## 4 Isolation of exosomes from body fluids

Exosomes in various body fluids are derived from diverse cellular origins and play essential roles in many physiological and pathological processes. The expansion of the sources of exosomes highlights their biological and clinical significance across different systems. Exosomes in blood originate from a wide range of cells, including blood cells, endothelial cells, and cells from solid organs. These exosomes carry a plethora of molecules, including immune modulators, signaling molecules, and genetic material. They are critical in regulating systemic functions such as inflammation, blood clotting, and immune surveillance [[66], [67], [68]]. In the realm of disease, blood-derived exosomes are pivotal for understanding and diagnosing cardiovascular diseases, cancers, and infectious diseases [69,70]. They can act as liquid biopsy agents, providing a snapshot of the tumor or disease environment without the need for invasive procedures [71].

Urinary exosomes primarily come from the renal epithelial cells. They provide insights into the cellular health and activities within the urinary tract and kidneys [72]. Because these exosomes can reflect changes in the renal cells due to disease, they are invaluable for the early detection of renal pathologies, including acute kidney injury and chronic kidney disease. Their non-invasive accessibility makes them ideal for ongoing monitoring of disease progression or response to therapy [73].

Exosomes in saliva are produced by salivary glands and other cells in the oral cavity. They offer a window into both oral and systemic health, encompassing potential markers for diseases such as Sjögren's syndrome, oral cancers, and even systemic conditions like diabetes [74]. Salivary exosomes can be easily collected, making them a convenient source for regular health screenings and disease monitoring.

CSF exosomes are derived from neural cells, including neurons and glia. They are particularly valuable in neurology, providing critical information about neurodegenerative diseases such as Alzheimer's disease, multiple sclerosis, and traumatic brain injuries [75]. CSF exosomes can carry biomarkers related to disease pathology and neural cell health, offering a means to diagnose or track neurological conditions through spinal taps [76].

Exosomes in breast milk, originating from mammary epithelial cells, play roles in maternal-infant communication. They carry immune-related molecules that help shape the infant's immune system and gut microbiome. Studying these exosomes helps understand maternal factors that influence infant immunity and development, offering potential interventions to boost neonatal health [77].

Exosomes from the lacrimal glands and ocular surface contain specific markers relevant to eye health. They are being explored for their role in diagnosing and understanding diseases like dry eye syndrome, ocular allergies, and even more severe conditions such as diabetic retinopathy or ocular cancers [78].

Exosomes in sweat, which originate from sweat glands, provide information about metabolic processes and stress responses. They hold promise for use in sports and exercise science to optimize performance and recovery, as well as in dermatological studies to explore conditions affecting sweat gland function or skin health [79].

**Fig. 6:**
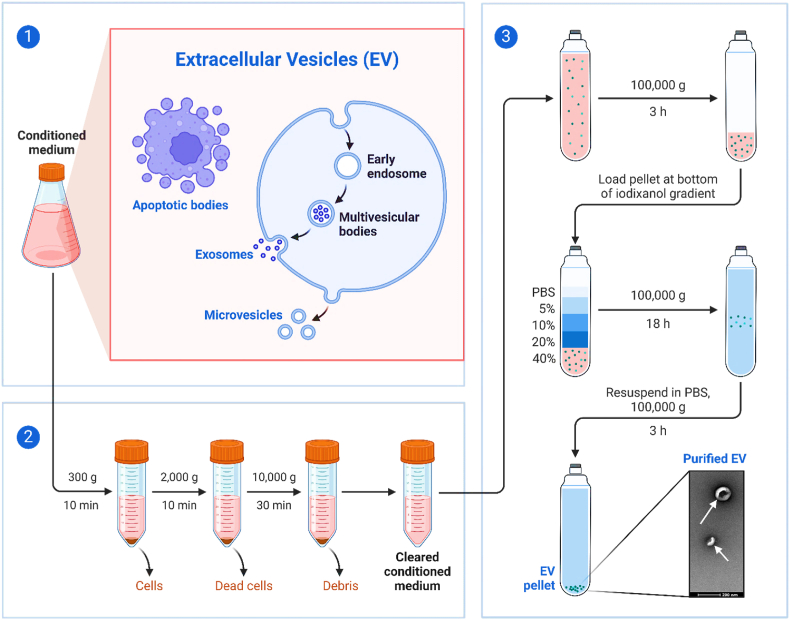
Ev separation by density gradient ultracentrifugation (created with BioRender.com) [84].

Isolating exosomes from serum involves several key steps to ensure purity and functionality of these small extracellular vesicles. The process typically starts with the collection of serum from blood samples, followed by differential centrifugation to remove cells and large debris. This is often followed by ultracentrifugation, which helps to pellet the exosomes. To further purify the exosomes, techniques such as density gradient centrifugation or size exclusion chromatography can be employed. These steps help to separate exosomes from other proteins and particles based on their size and density. Finally, the isolated exosomes can be analyzed for their protein, lipid, and RNA content, confirming their identity and assessing their potential for research or therapeutic applications [80,81]. Isolating exosomes from serum offers several advantages, such as providing valuable insights into disease diagnostics and biomarker discovery due to the rich content of proteins, RNA, and lipids that reflect the physiological state of their cells of origin. Serum-derived exosomes are particularly useful for non-invasive liquid biopsies, allowing for continuous monitoring of disease progression or response to therapy. However, challenges persist, including the heterogeneity of exosome populations, which complicates their isolation and analysis [82]. Additionally, the presence of serum proteins similar in size and density to exosomes can lead to contamination, requiring meticulous technique and advanced purification strategies to ensure sample purity and the reliability of subsequent analyses. Exosomes in body fluids reflect the physiological state and health of various tissues and organs. They are indispensable for modern diagnostics and personalized medicine, offering non-invasive ways to assess health, diagnose diseases, and monitor treatment responses across a broad spectrum of medical disciplines [83]. Table 1 & Fig. 6 outlines the sources, significance, and applications of exosomes from various body fluids, providing a clear and concise overview of their role in health and disease diagnostics and research ( Table 1, Table 2).

**Table 1:** The sources, significance, and applications of exosomes from various body fluids.

Body Fluid	Origin of Exosomes	Biological Significance	Applications
Blood	Blood cells, platelets, endothelial cells, cancer cells	Systemic communication, immune modulation, disease biomarkers	Liquid biopsy for cancers, monitoring of inflammatory and autoimmune diseases
Urine	Renal cells, urinary tract cells	Indicates kidney and urinary system health	Non-invasive diagnosis and monitoring of kidney diseases
Saliva	Salivary glands, oral mucosa	Reflects oral and systemic health	Diagnostics for oral diseases and systemic conditions like diabetes and Sjögren's syndrome
Cerebrospinal Fluid (CSF)	Neural cells (neurons, glial cells)	Reflects neurological health and disease	Diagnosis and research of neurodegenerative diseases, monitoring neurological damage
Breast Milk	Mammary gland epithelial cells	Immune system development in infants	Studies on infant health and development, understanding maternal impact on infant immunity
Tears	Lacrimal glands, ocular surface cells	Indicator of ocular health	Early detection and monitoring of ocular diseases, research into systemic diseases affecting the eyes
Sweat	Sweat glands	Reflects metabolic and physiological responses	Used in sports medicine for performance optimization, dermatological research on skin conditions

**Table 2:** Comparison on isolation methods of exosomes.

Isolation Method	Principle	Efficiency and Purity	Advantages	Disadvantages	Suitability for Different Types of Fluids
***Ultracentrifugation***	Objects in the tube remain in the location of the medium with a same density after centrifugation in a dense media.	High purity but may have variable efficiency depending on conditions.	The gold standard, is high purity, with no need for specific reagents. Less likely to contaminate with additional isolation reagents. Works well for large-scale preparations.	Time-consuming, requires expensive equipment, potential damage to exosomes, low yield, not portable, risk of mechanical damage to the sample. Protein aggregates, making it unsuitable for diagnosis of small volumes.	Ideal for research settings with available equipment.
***Size-based Isolation***	It uses a filter membrane with a molecular weight cut-off or size-exclusion limit that is specified. After adding to porous materials, substances are eluted out in accordance with their particle size.	Moderate to high purity; efficiency varies with technique.	Relatively fast, scalable, gentle on exosomes, and does not require highly specialized equipment. Isolation gives products fast to prepare and keep exosomes in their native states.	May require further purification steps. Samples of moderate purity can be tested. Expensive equipment required for further enrichment technique.	Broad applicability across various fluids including blood, urine, and saliva.
***Immunoaffinity Capture***	It is based on the unique way that immobilized antibodies (ligands) and exosome markers bind.	Very high purity, efficient capture of specific exosome populations.	High specificity, can isolate specific subpopulations of exosomes. Ideal for isolating exosomes from certain sources; highly pure exosomes, simple to use, and free of chemical contamination.	Costly, dependent on the availability of high-quality antibodies, and time-consuming.	Best for fluids where specific exosomal markers need to be targeted, like blood.
***Polymeric Precipitation***	Precipitation of polymers with big particles eluting earlier. Water-excluding polymers with high hydrophilicity can change how soluble exosomes are.	Moderate purity, and high efficiency in terms of yield.	Simple, quick, and able to handle large volumes effectively. High efficiency and can handle both small and big sample volumes.	Co-precipitates other proteins and particles, can interfere with subsequent assays. Polymeric pollutants, extracellular vesicles, and protein aggregate contamination. Long processing times and difficult cleanup procedures that affect quantification and downstream analysis.	Effective for a variety of fluids, especially when large volumes need processing.
***Microfluidic Techniques***	Based on several criteria, such as size and density, immunoaffinity.	High purity and efficiency; highly specific.	High specificity, minimal sample requirements, integration of multiple steps into a single design. This approach is incredibly productive, economical, portable, and simple to automate and integrate with the diagnosis.	Requires specialized equipment and expertise, and can be costly to design and operate.	Ideal for precise diagnostic work, suitable for small sample volumes like CSF.

## 5 Methods of exosome isolation

Exosomes are small vesicles present in various bodily fluids, and their isolation is challenging due to their minute size. While ultracentrifugation has traditionally been favored for processing large sample volumes [85], it can introduce contaminants like proteins and lipoproteins into exosome samples, impacting their analysis and functional exploration. Recognizing that no single technique is universally effective for all sample types, researchers have investigated various methods that capitalize on the distinct physical and biochemical characteristics of exosomes. Currently, six primary methods have been established: high-speed centrifugation, ultrafiltration, immunoaffinity capture, polymer precipitation through charge neutralization, size-exclusion chromatography, and microfluidics. Each technique offers specific advantages and limitations [[86], [87], [88]]. This broad perspective helps improve exosome isolation for different uses and sparks ideas for creating new tools and methods for more effective isolation [[89], [90], [91], [92]].

### 5.1 Pioneering exosomes studies based on differential ultracentrifugation

**Fig. 7:**
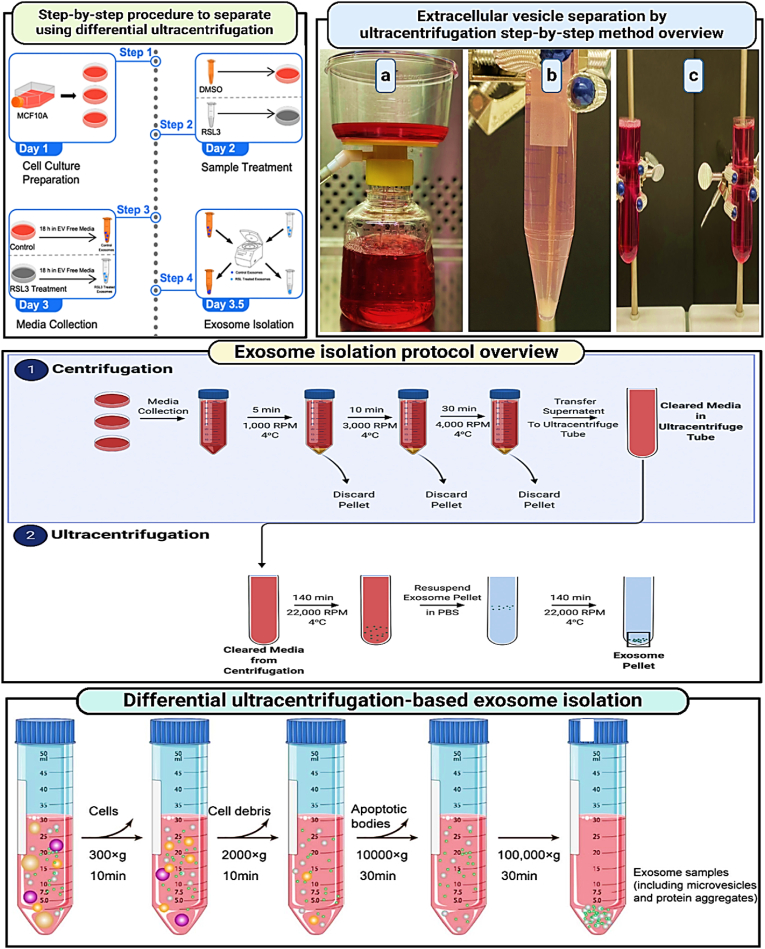
**Procedure for isolating extracellular vesicles via ultracentrifugation** (Shared under the Creative Commons CC BY 4.0 license, source [106], Copyright 2021 by the Authors); **Methodology for exosome isolation using differential ultracentrifugation** (Shared under the Creative Commons CC BY 4.0 license, source [107], Copyright 2020 by the Authors).

Differential ultracentrifugation, also known to be as the pelleting method or simple ultracentrifugation, is a widely used approach for separating exosomes, accounting for 45.7 % [[93], [94], [95], [96]] of reported strategies. The concept behind this method is straightforward: it separates different components of a fluid sample by their density, size, and shape under varying centrifugal forces [97,98]. This technique was first employed by Johnston in 1987 to extract exosomes from reticulocyte tissue cultures. Later, in 2006, Thery and colleagues refined the method by applying a range of centrifugal forces. The process often begins with a low-speed centrifugation step to remove larger particles, followed by several higher-speed cycles to clear out cellular debris, any protein aggregates and apoptotic bodies, thereby isolating more clean exosome samples [99,100]. The above method is scalable for large-volume preparations, despite the common use of ultracentrifugation tubes with relatively small capacities. Due to its simplicity, minimal technical requirements, and compatibility with large volumes without needing complex sample preparation, differential ultracentrifugation has become a staple method for isolating exosomes from various biological fluids like cell culture media, serum, and cerebrospinal fluid over the last three decades. However, the diverse nature of extracellular fluids means that under specific centrifugal forces, all components—including undesirable ones like protein aggregates—can precipitate, leading to low-purity exosome samples that may hinder further functional analyses [[101], [102], [103]]. For instance, Paolini and colleagues found that exosomes isolated from the blood of multiple myeloma patients using this method showed poor biological functions in comparison to more purified samples, which had a significant impact on cellular processes in their studies [[102], [103], [104], [105]]. To enhance the purity and efficiency of exosome isolation, researchers have developed various centrifugation techniques over the years, exploring different physical properties. One such improvement is density-gradient centrifugation, which separates particles based on their density, offering a more refined approach to isolation of exosomes. Multiple cycles of centrifugation are performed in differential ultracentrifugation with centrifugal forces of about 300×*g* up to 100,000×*g*. Pellets including cells, cell debris are removed after each centrifugation, the supernatant is collected for next step of ultracentrifugation. Ultimate stages of centrifugation at 100,000×*g* is followed by collection of exosomes containing pellets and contaminant proteins and subsequent supernatant removal. All stages of centrifugation were performed at 4 °C. (Fig. 7).

### 5.2 High quality exosome isolation by isopycnic & moving-zone density-gradient ultracentrifugation

Linderstorm-Lang in 1937, made a key discovery in density-gradient centrifugation field: similar density objects stay suspended in a medium that matches their density after centrifugation [108,109]. This principle has been especially useful in hematological research for separating different blood cell types, which vary in density. The same principle applies to isolating extracellular components such as exosomes. A typical density-gradient ultracentrifugation [110,111] process starts by layering a biocompatible medium with varying densities, like iodixanol or sucrose, in a tube with the densest layer at the bottom and decreasing upwards. The sample is then placed on top of this gradient and centrifuged at high speeds (e.g., 100,000×*g* for 16 h). During centrifugation, components like exosomes, apoptotic bodies, and protein aggregates settle at their respective densities, creating clear separation. For example, protein aggregates gather at the bottom, while exosomes are found in layers with densities between 1.10 and 1.18 g/mL. This method yields highly pure exosome samples, making it more effective than simpler techniques like differential ultracentrifugation or one-step precipitation kits. As a result, density-gradient ultracentrifugation has become increasingly popular, now representing about 11.6 % of exosome separation methods used today. However, standard density-gradient centrifugation cannot separate extracellular vesicles like microvesicles from exosomes if they share similar densities. To overcome this, moving-zone or rate zonal centrifugation has been employed. This method uses a gradient medium that is lighter than all components in the sample. After centrifugation, particles separate based on both their size and density, allowing for the isolation of different types of vesicles.

Despite these advancements, ultracentrifugation has limitations. For instance, the gradient method can only handle small volumes due to its narrow loading zone. The equipment is costly and requires skilled operators, particularly for gradient techniques. Moreover, the intense centrifugal forces can alter the exosomes' structure and function, which is detrimental for applications like drug development. In response, alternative methods like ultrafiltration and size-exclusion chromatography have been developed. These newer approaches, along with commercial exosome separation kits, offer simplified and efficient solutions, which we will explore further in the following sections.

**Fig. 8:**
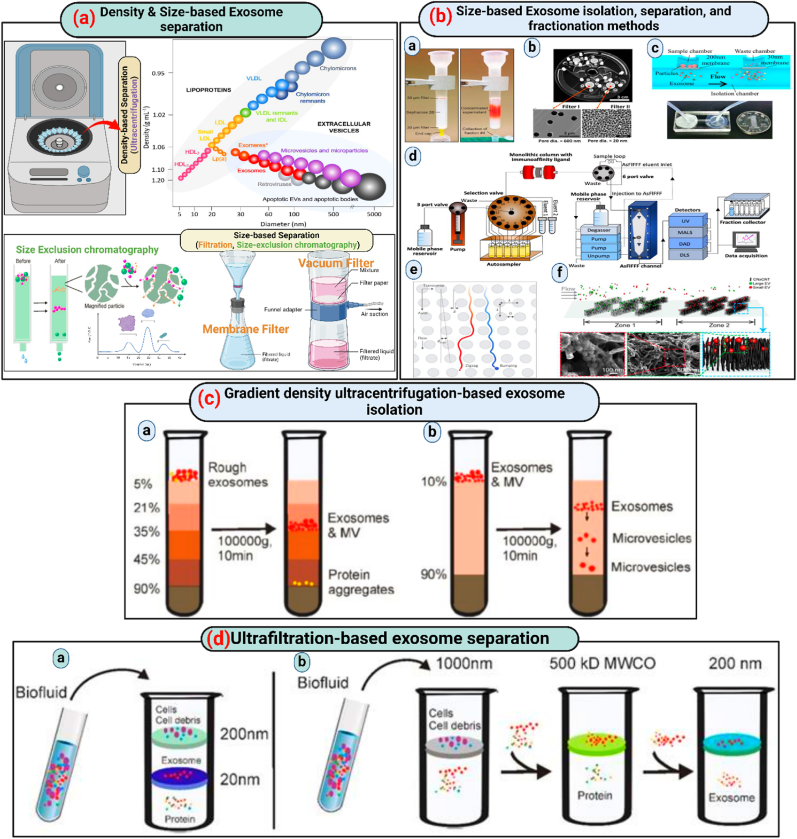
**(a) Approaches for separating exosomes based on density and size** (Summarized from Refs. [[112], [113], [114], [115]]); **(b) Various methods for isolating and fractionating EVs according to size: a) Exosome extraction from tumor cell culture media using a Mini-SEC column** (Adapted with authorization from Ref. [116], copyright 2019 by Elsevier); **b) Isolation of urinary EVs utilizing Exodisc** (Adapted with authorization from Ref. [117], copyright 2017 by the American Chemical Society); **c) Double filtration microfluidic device for urinary EV isolation** (Shared under the Creative Commons Attribution CC BY license, source [118], copyright 2017 Springer Nature); **d) Combining immunoaffinity chromatography with asymmetrical flow field-flow fractionation to isolate EV subpopulations from human plasma** (Adapted with authorization from Ref. [119], copyright 2020 by the American Chemical Society under the Creative Commons Attribution CC BY license); **e) Lateral displacement arrays for exosome separation** (Adapted with authorization from Springer Nature, copyright 2016); **f) Capture of size-based EVs from cell culture using three-dimensional carbon nanotube arrays** (Adapted with authorization from Refs. [120,121], copyright 2020 by the American Chemical Society); **(c) Diagram illustrating exosome isolation by gradient-density ultracentrifugation** (Shared under the Creative Commons Attribution CC BY 4.0 license, source [122], Copyright © 2020 by the Authors); **(d) Exosome separation via ultracentrifugation** (Shared under the Creative Commons Attribution CC BY 4.0 license, source [122], Copyright © 2020 by the Authors).

In Fig. 8, (A) Addition of medium in isopycnic density-gradient ultracentrifugation is in layers of decreasing density from bottom to top. Apoptotic body, exosomes and aggregates of proteins like extracellular components Obtain a stationary position in a medium with a density comparable to every component in this kind of extended centrifugation. Ultracentrifugation with an isopycnic gradient is unable to separate substances, with similar buoyant density to exosomes because it depends only on the density difference between solutes in samples. (B) Dual gradient medium sections are usually seen in moving-zone gradient ultracentrifugation normally. The medium in the top layer has lower density than all other solutes in the sample and the bottom layer has high-density. The gradient medium density is lower than all the solutes, so all the solutes after centrifugation are separated based on mass/size, not only on density, allowing for the separation of vesicles of varying size but of comparable density.

### 5.3 Size-based isolation techniques

In 1955, Grant H.L and Colin R.R introduced size-exclusion chromatography (10.13039/100023562SEC), a technique for separating solutes based on molecular weight [103]. This method involves passing a liquid sample through a column filled with porous particles. Smaller molecules that can enter these pores are slowed down, while larger molecules that cannot enter are eluted more quickly. Initially using starch and water, the materials used in SEC have evolved over the decades to Agarose (Sepharose), Dextran polymer (Sephadex), and polyacrylamide (Sephacryl or BioGel) which are sophisticated porous materials.

SEC has been effectively adapted for exosome isolation due to the physical similarities between exosomes and liposomes. This method has been harnessed by companies like iZON with their qEV kits and Hansa Biomed with PURE-EVs to specifically isolate exosomes. The gentle nature of SEC, which uses gravity flow rather than force, helps preserve the biological activity and structural integrity of exosomes, with elution buffers mimicking physiological conditions to maintain functional states. SEC offers practical benefits including minimal sample volume requirements, compatibility with various fluid types without pre-treatment, and efficiency that saves time and labor. It also allows for the isolation of specific exosome subpopulations through selective porous materials, ensuring minimal sample loss and high yield, making it popular for both research and clinical applications. Despite these advantages, SEC faces challenges such as potential contamination with similar-sized particles like protein aggregates. To address this, strategies combining SEC with ultrafiltration have been proposed to enhance purity and maintain functionality, showing promise for improving the quality of isolated exosomes. Illustrative details include Fig. 8; (A) A tandem-configured microfilter setup traps apoptotic bodies, cell debris, and most microvesicles with a 200-nm membrane as extracellular fluids pass through filters with defined size-exclusion limits. Fig. 8; (B) Sequential ultrafiltration processes involve removing larger particles through a 1000-nm filter, smaller particles such as free proteins up to 500-kD through a second filter, and finally isolating exosomes under 200 nm with a third 200-nm filter. In SEC, molecular separation by size occurs as a solution passes through a stationary phase of porous resin particles where larger particles bypass directly and smaller ones navigate through the resin, leading to effective size-based separation.

### 5.4 Polymeric precipitation technique

**Fig. 9:**
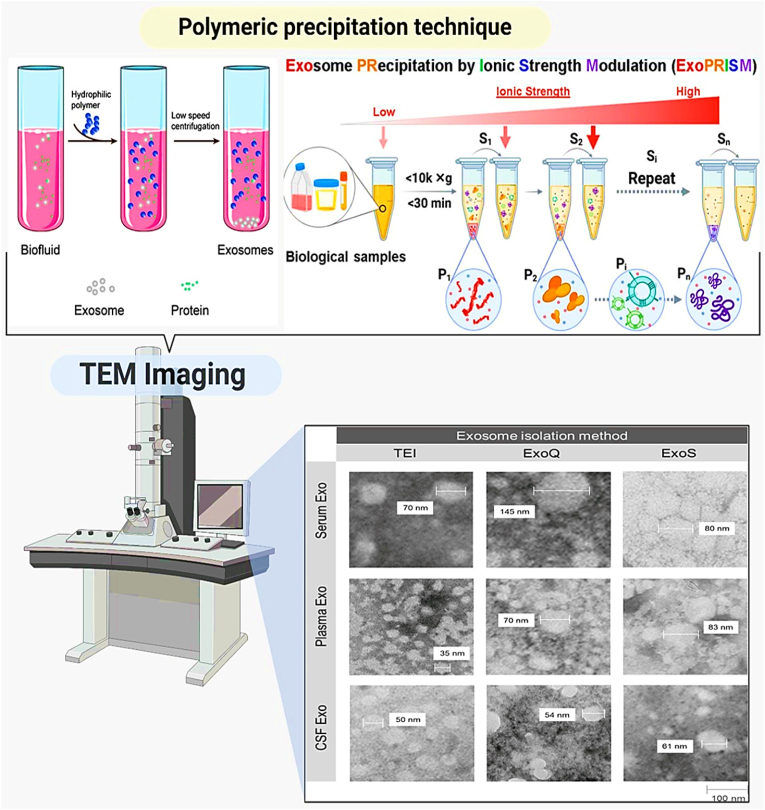
**Exosomal morphology determination by pooled TEM negative staining.** Two precipitation-based (TEI and ExoQ) and one column-based method (ExoS) for exosome isolation from distinct biofluids (Adapted from Ref. [123]).

The polymeric precipitation technique is a commonly used method for isolating exosomes that involves adding a water-soluble polymer, such as polyethylene glycol (PEG), to the exosome-containing sample. This polymer induces the precipitation of exosomes by decreasing the solubility of vesicles in the solution. Once the polymer is added, the mixture is incubated and then centrifuged to collect the precipitated exosomes as a pellet. This method is favored for its simplicity and rapid processing, allowing for the isolation of exosomes from large volumes of fluid efficiently. It is also less equipment-intensive compared to ultracentrifugation. However, one of the main drawbacks of this technique is the potential co-precipitation of other proteins and particles, which can lead to less pure exosome preparations. Additionally, the presence of polymers can sometimes interfere with subsequent analytical and diagnostic assays, requiring careful consideration and potentially additional purification steps post-isolation (Fig. 9).

### 5.5 Immunoaffinity capture

**Fig. 10:**
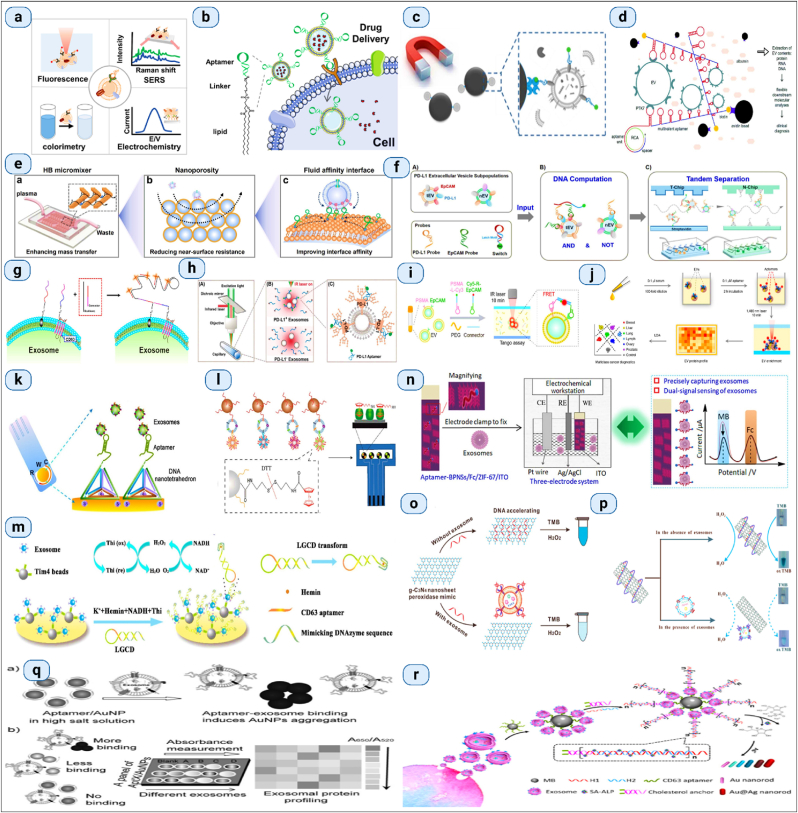
**Aptamer-based immunoaffinity capture for EV analysis includes: (a) Aptamer-based evaluation of EVs. (b) Therapeutic applications using aptamer-functionalized EVs.** (Adapted from Reference [134]); Various **aptamer-based methods for EV isolation: (c) Exosome isolation via immunomagnetic beads** (Adapted from Reference [135], Copyright 2019 by the American Chemical Society); **(d) EV isolation using RCA-induced multivalent aptamers** (Adapted from Reference [136], Copyright 2021 by The Royal Society of Chemistry); **(e) Enhancing affinity interactions for EV detection using a fluid nanoporous microinterface, termed FluidporeFace** (Adapted from Reference [137], Copyright 2022 by Elsevier); **(f) Isolation of the PD-L1 EV subpopulation using DNA computation mediated microfluidic tandem separation** (Adapted from Reference [138], Copyright 2022 by the American Chemical Society). **Fluorescence detection of EVs via Aptamers includes: (g) Enhanced exosome detection by RCA** (Adapted from a 2020 publication by the American Chemical Society); **(h) Quantification of Exosomal PD-L1 using a PD-L1 aptamer combined with thermophoresis** (Adapted from Reference [139], Copyright 2020 by Wiley-VCH Verlag GmbH & Co. KGaA, Weinheim); **(i) Detection of tumor-derived EVs via dual-aptamer induced FRET** (Adapted from Reference [140], Copyright 2022 by Wiley-VCH Verlag GmbH); **(j) EV protein profiling employing multiple aptamers and thermophoresis** (Adapted from Reference [141], Copyright 2019 by Springer Nature). **Detection of EVs using an electrochemical Aptamer-based approach includes: (k) Quantification of exosomes using NTH-assisted electrochemical aptasensors** (Adapted from Reference [142], Copyright 2017 by the American Chemical Society); **(l) Analysis of protein subpopulations within exosomes** (Adapted from Reference [143], Copyright 2020 by the American Chemical Society); **(m) ExoPCD-chip: A two-stage microfluidic platform** (Adapted from Reference [144], Copyright 2018 by the American Chemical Society); **(n) Recalibrating aptasensor** (Adapted from Reference [145], Copyright 2020 by the American Chemical Society). **Colorimetric detection of EVs via Aptamer-based methods includes: (o) Detection of exosomes using GO-mediated colorimetric methods** (Adapted from Reference [146], Copyright 2017 by the American Chemical Society); **(p) S-SWCNTs-based colorimetric detection of exosomes** (Adapted from Reference [147], Copyright 2017 by Elsevier B.V.); **(q) Exosome protein profiling through salt-induced aggregation of aptamers** (Adapted from Reference [148], Copyright 2017 by Wiley-VCH Verlag GmbH & Co. KGaA, Weinheim); **(r) Multicolor-based colorimetric detection technique** (Adapted from Reference [149], Copyright 2019 by the American Chemical Society).

Immunoaffinity capture is a targeted approach for isolating exosomes that exploits specific interactions between antibodies and exosomal surface proteins [124,125]. This technique involves using antibodies that are specific to exosomal markers, such as CD63, CD9, and CD81, which are conjugated to solid support like beads or a plate. The sample containing exosomes is passed over or mixed with these antibody-coated supports, allowing the exosomes to bind selectively based on their surface proteins [126]. After binding, the exosomes can be eluted from the support, resulting in a highly specific and pure population of exosomes [127]. Immunoaffinity capture is particularly valued for its high specificity and ability to isolate exosome subpopulations from complex biological fluids, making it ideal for applications requiring high purity and for studies focusing on specific exosomal populations [128,129]. However, it is typically more costly and time-consuming than other isolation methods, and the availability of high-quality antibodies can be a limiting factor [130,131]. Antibodies are immobilized onto solid matrices which recognize exosome-specific markers at first, after which extracellular fluids containing exosomes are incubated with antibody-conjugated solid matrices, by which exosomes get enriched onto such solid matrices [132,133]. An additional elution step in required for collection of free exosomes. (Fig. 10).

### 5.6 Next generation immunoaffinity approach based on chemical antibody

While products generated from antibodies offer various advantages for applications such as exosome isolation, their high development and production costs, along with their perishability, limit their practical use, especially on a large scale. To address these challenges, one alternative is to utilize more cost-effective and stable substitutes like aptamers. Aptamers are short, single-stranded DNA or RNA molecules that bind to their specific targets with high affinity and specificity, akin to antibodies. However, they boast several benefits over traditional antibodies: they can be synthesized chemically in the lab, resulting in minimal batch-to-batch variations; they are easier to scale for various needs; they have longer shelf lives; they exhibit little or no immunogenicity; they are cheaper to produce; and they can be chemically modified to enhance their binding properties [150].

**Fig. 11:**
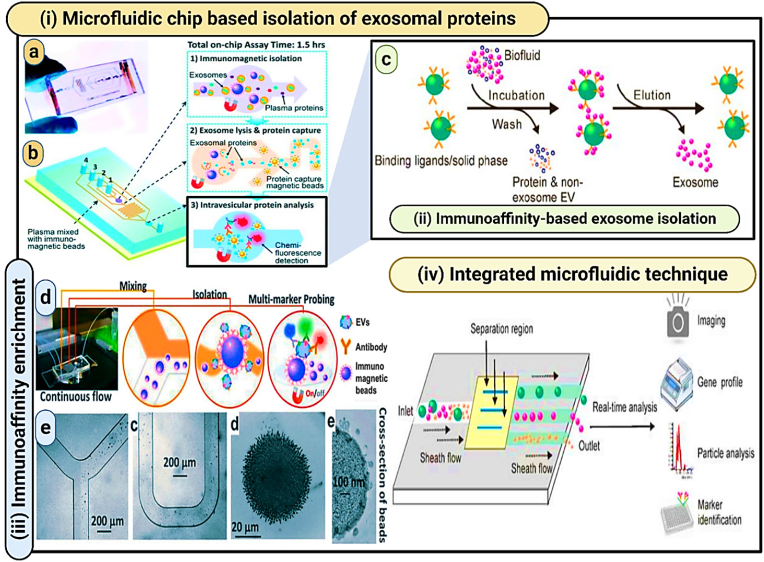
**(i) Integrated microfluidic technology for the isolation of circulating exosomes** (Reproduced with Creative Commons CC BY 4.0 license from Ref. [154], Copyright © 2014 by the Authors); **(ii) Diagram illustrating immunoaffinity-based exosome isolation** (Reproduced with Creative Commons CC BY 4.0 license from Ref. [122], Copyright © 2020 by the Authors); **(iii) Techniques for immunoaffinity enrichment of exosomes** (Reproduced with Creative Commons CC BY 4.0 license from Ref. [155], Copyright © 2016 by the Authors); **(iv) Analysis and isolation of exosomes using an integrated microfluidic approach** (Reproduced with Creative Commons CC BY 4.0 license from Ref. [122], Copyright © 2020 by the Authors).

In recent years, various aptamer-based platforms for isolating exosomes have been developed. These platforms offer a practical approach to isolate exosomes based on immunoaffinity and simplify the process of obtaining natural exosomes [151]. The function of aptamers is heavily influenced by their tertiary structure, which itself depends on factors such as temperature, ionic strength, and the buffer system used. By fine-tuning these conditions—like adjusting the types of salts and key ions such as Mg^2+^ and K^2+^ researchers can modify the three-dimensional structure of aptamers. This adjustment allows for the mild remodulation of their binding capacity, enabling the release of captured exosomes while preserving their native structure and biological functionality [152,153]. Conformational complementarity is how aptamers bind their target. Aptamers undergo change in shape and release their target molecules by adjustment of key factors of the buffering system like type of salt attached, change in ionic strength (Fig. 11).

### 5.7 Microfluidic technique

**Fig. 12:**
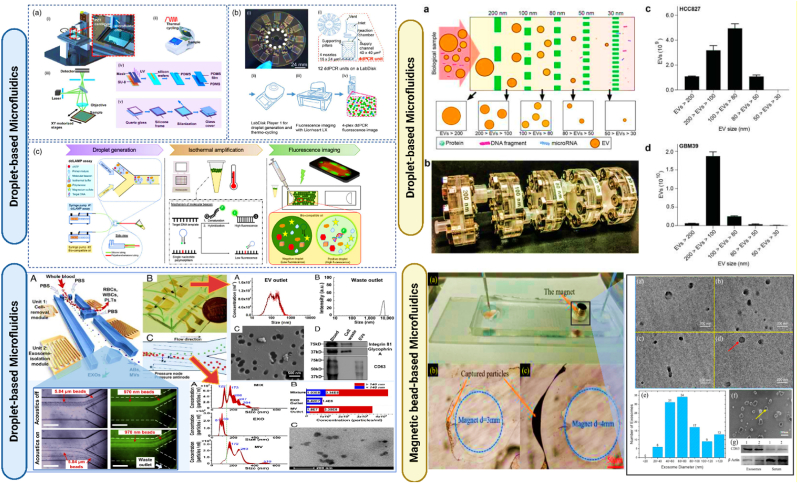
**Microfluidic Devices for Exosome Isolation: Droplet-based microfluidics**: **(a)** Schematic diagrams of a microfluidic printing droplet digital polymerase chain reaction setup (MIP-ddPCR setup). (Reproduced with permission from Ref. [156], Copyright 2019, Elsevier); **(b)** Workflow for an automated four-plex digital droplet PCR (ddPCR) in a LabDisk. (Reproduced under terms of the CC-BY license from Ref. [157], Copyright 2021); **(c)** Methods for performing ddLAMP using an assay featuring MB as a sequence-specific probe for single nucleotide polymorphisms (SNP) detection. (Reproduced with permission from Ref. [158], Copyright 2022, American Chemical Society.); **Size-based isolation of EVs using the ExoTIC device** (Reproduced with Creative Commons CC BY 4.0 license from Ref. [159], Copyright © 2017 by the Authors); **Integrated Acoustofluidic device** for isolating exosomes (Reproduced with Creative Commons CC BY 4.0 license from Ref. [160], Copyright © 2017 by the Authors); **Magnetic Bead-Based Microfluidics for Enhanced Exosome isolation** (Reproduced with Creative Commons CC BY 4.0 license from Ref. [161], Copyright © 2017 by the Authors).

**Fig. 13:**
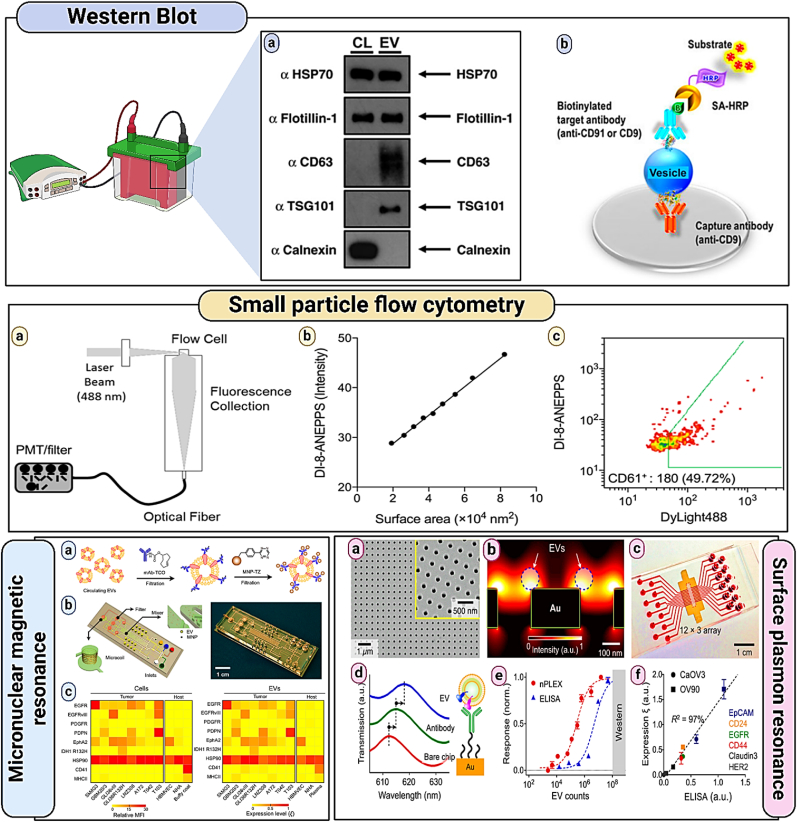
**Conventional methods for analyzing EV proteins include: (a) Western blotting: This technique involves the separation of EV protein lysates using SDS-PAGE (sodium dodecyl sulfate polyacrylamide gel electrophoresis), followed by the transfer of specific EV protein targets (such as Flotillin-1, HSP-70, and CD61) onto a membrane for immunoblotting** (Adapted from Reference [164], Copyright 2015 by Taylor & Francis Ltd.); **(b) ELISA (Enzyme-linked Immunosorbent Assay): In this method, vesicles or lysates are attached to a solid support that has been pretreated with an immobilized capturing antibody. A target antibody then detects the EV protein targets in a “sandwich” assay format** (Adapted from Reference [165], Copyright 2015 by Nature Publishing Group); **Small particle flow cytometry** (Shared under the Creative Commons CC BY 4.0 license from Reference [166], Copyright © 2016 by the Authors); **Micronuclear magnetic resonance** (Shared under the Creative Commons CC BY 4.0 license from Reference [167], Copyright © 2012 by the Authors); **Surface plasmon resonance** (Shared under the Creative Commons CC BY 4.0 license from Reference [168], Copyright © 2014 by the Authors).

The microfluidic technique for exosome isolation utilizes the advanced capabilities of microfluidics technology. Initially, a biological sample, such as blood or urine, is introduced into the microfluidic device. This device contains a network of microscale channels that can precisely manipulate the fluid flow. As the sample flows through these channels, exosomes are separated based on specific properties—size, density, or biochemical markers. For size-based isolation, the channels are designed to allow smaller particles to flow through while capturing larger ones; for biochemical isolation, the inner surfaces of the channels are often coated with antibodies that specifically bind to proteins on the exosome surface, such as CD63, CD9, or CD81. Once the exosomes are captured, a series of washing steps follow to remove unbound material and impurities. The final step involves the elution of captured exosomes from the channels, which are then collected for further analysis. This system not only allows for the selective isolation of specific exosome subpopulations but also significantly enhances the efficiency and specificity of the process. The reduced volume requirements and the ability to process samples quickly are substantial benefits, particularly for clinical applications where sample volume and time are critical. Moreover, microfluidic devices can maintain the structural integrity of the isolated exosomes, ensuring high purity and functionality for downstream analyses. However, despite these advantages, the complexity of designing and operating these devices and the need for specialized knowledge can make microfluidics less accessible for routine use outside specialized settings (Fig. 11, Fig. 12 & Table 3) [122,154,155,[156], [157], [158], [159], [160], [161], [162], [163]] (see Fig. 13).

**Table 3:** Overview of microfluidic devices for exosome isolation: Mechanisms, advantages, and key features.

Device Type	Mechanism	Advantages	Key Features
***Droplet-Based Microfluidics***	Encapsulates exosomes within fluid droplets for individual analysis.	High throughput, minimal cross-contamination, and precise single-exosome analysis.	Ideal for studies requiring high isolation purity and analytical precision.
***Size-Based Microfluidic Channels***	Uses microfabricated channels that separate particles based on size.	Simple setup, effective at removing larger vesicles and debris, no chemical reagents needed.	Useful in applications where size distinction is crucial for sample purity.
***Immunocapture Microfluidic Devices***	Incorporates specific antibodies to capture exosomes with corresponding antigens.	High specificity, can isolate exosomes from specific cell types or conditions.	Best for targeted exosome recovery, especially in clinical diagnostics involving specific markers.
***Acoustic Microfluidics***	Utilizes ultrasonic waves to manipulate and separate exosomes based on density and compressibility.	Gentle on samples, preserving exosome integrity, scalable for larger volumes.	Suitable for applications needing non-invasive and label-free separation methods.
***Centrifugal Microfluidics***	Employs centrifugal force to effect separation by size and density on a spinning disc.	Integrates several laboratory functions, automates processes, reduces manual handling.	Known as “lab-on-a-disc,” it's effective for processing multiple samples simultaneously.
***Electrokinetic Microfluidics***	Applies electric fields to move and separate exosomes based on their charge and size.	Highly precise, allows for the separation of exosomes from other similarly sized particles.	Appropriate for detailed studies of exosome properties influenced by electric fields.
***Nano-Interferometric Detectors***	Integrates nano-interferometric sensors within a microfluidic device to detect exosomes by refractive index.	Provides label-free detection and quantification, no need for fluorescent or enzymatic labels.	Excellent for real-time monitoring and quantifying exosome concentration in research and clinical settings.
***Integrated Microfluidic Systems***	Combines multiple separation techniques such as immunocapture and size exclusion within a single device.	Enhances purity and yield through sequential processing, offers flexibility in isolation protocols.	Ideal for comprehensive studies where multiple isolation phases are needed for optimal purity.
***Magnetic Bead-Based Microfluidics***	Uses magnetic beads coated with antibodies specific to exosomal markers in a magnetic field.	Efficient capture and concentration, easy scale-up, and sample processing.	Highly effective for isolating specific exosome populations, especially useful in scalable clinical diagnostics.

## 6 Exosomal protein analysis

### 6.1 Protein analysis of extracellular vesicles by conventional methods like ELISA, western blot

Immunoblotting, or western blotting, is a popular protein analysis method used in many fields of molecular biology. In when evaluating EV protein [170], Western blotting is perhaps the most often employed method to show the existence of target proteins that are purportedly connected to EVs. During this procedure, purified vesicle preparations produced using the most recent gradient ultra gold standard buffered lysis solutions may be used to treat centrifugation which are composed of protease inhibitors and denaturants [171,172]. The peptide Next, sodium dodecyl sulfate is used to separate the lysates. Prior to using SDS-PAGE (polyacrylamide gel electrophoresis), being moved to a membrane in order to be immunoblotted using particular targets for proteins [173,174]. Although the strategy has a lengthy processing and preparation period (>10 h), Western Blotting might offer helpful details on the size. Enzyme-linked immunosorbent assay (ELISA), which may be performed in a variety of test formats, is another well-established method for quantifying proteins, similar to Western blotting. Purified vesicle preparations or lysates might be placed directly to a solid substrate that has been prepared with an immobilized capturing antibody in the particular “sandwich” configuration. Captured vesicular targets are subsequently exposed to another detecting antibody. Although the necessity for two noninteracting antibodies increases the specificity of detection, it complicates the process of creating novel assays and carrying out multiplexed, simultaneous measurements. The limits of detection for ELISA and Western blotting are comparable. However, ELISA may be scaled up for higher-throughput tests and can be much faster than Western blotting [175].

### 6.2 Small particle flow cytometry

Based on light scattering and fluorescence activation, flow cytometry is a potent technique for characterizing single large particles (such as cells or larger micrometer-size entities); however, conventional flow cytometry has limited sensitivity and resolution to detect small particles that have a diameter <500 nm [176,177]. Moreover, it has a strong optical background because sheath fluids include tiny particles (200 nm or less). Many small EVs may be overlooked or undercounted when using traditional flow cytometry for EV quantification because many small vesicles must be lit concurrently to trigger a count; as a result, they are tallied as a single event. The term “Swarm Theory” refers to this latter phenomenon [[178], [179], [180]]. Multiple vesicles have been bound by micrometer-sized latex beads in order to modify standard flow cytometry for EV profiling. After then, fluorescent antibodies are used to dye the bound EVs, and their protein markers are examined. Nevertheless, this method cannot distinguish between various vesicular subsets and is not capable of profiling a single vesicle, which might lead to the loss of distinguishing characteristics [181,182].

### 6.3 Micronuclear magnetic resonance

Particular magnetic nanoparticles (MNPs)-based magnetic sensing has drawn a lot of interest lately. Since most biological things inherently lack ferromagnetic background, native biological samples do not significantly interfere with such detection. When targeted with appropriate MNPs [183,184], even optically murky samples will appear clear to magnetic fields, achieving a strong contrast against the original biological backdrop. When MNPs are positioned in NMR magnetic fields, they produce local magnetic fields that alter the transverse relaxation rate of the nearby water molecules, enhancing the analytical signal in the case of magnetic detection based on nuclear magnetic resonance (NMR) [185,186]. NMR uses blood samples to directly identify bacteria and circulating tumour cells while reducing sample processing and increasing detection sensitivity.

However, due to the fact that these vesicles are one or two orders of magnitude smaller than tumour cells, it has proven extremely difficult to adapt NMR for EV detection. Shao et al. created a novel analytical technique especially for EV detection and protein profiling in order to close this size disparity [187]. This method involved labelling EVs with MNPs using a two-step bio-orthogonal click chemistry. By not significantly increasing the size of either the MNP or the antibody, this small molecule (<200 Da) labelling approach improved the efficacy of keeping the targeted vesicles free of unattached MNPs and antibodies. The abundance of EV biomarkers was then directly evaluated on-chip for targeted EVs using a microfluidic micronuclear magnetic resonance (μNMR). The new μNMR technology showed a much greater detection sensitivity, about 103-fold more sensitive than Western blotting and ELISA, when compared to existing protein technologies [187]. Shao et al. further evaluated EVs from glioblastoma multiforme (GBM) cell lines produced in culture using this integrated approach. In order to differentiate cancer-derived EVs from host cell-derived EVs, a four-GBM marker combination (EGFR, EGFRvIII, PDPN, and IDH1 R132H) was found. Comparative protein studies verified that EVs do, in fact, represent the protein profiles of their parental cells for the investigated markers.

### 6.4 Surface plasmon resonance

Surface plasmon resonance (SPR) is a unique sensing approach that enables quick, label-free characterization of EVs, given their tiny size. SPR is the collective oscillation of conduction electrons when incident light illuminates the metal-dielectric contact [188]. SPR sensing, in contrast to existing optical detection techniques that rely on time-sensitive fluorescent and chemiluminescent probes, may be deployed label-free and in real-time to detect changes in the local refractive index linked to biomolecular binding close to a metal-dielectric interface. Recently, Im et al. created the nanoplasmonic exosome (nPLEX) sensor, a novel SPR platform for EV protein identification [189]. Transmission SPR via periodic nanohole arrays is the basis for the sensing. For EVs, this transmission-type SPR has a number of benefits over the traditional reflection design. For EV profiling, this transmission-type SPR has two key advantages over the traditional reflection configuration: (1) the collinear transmission optical setup makes device miniaturisation easier, and (2) the probing depth (200 nm) can be easily adjusted to match EV size to improve detection sensitivity [190]. Through three-dimensional modelling studies, the nanohole design was further optimised to match the sensing range with the exosome mean diameter (about 100 nm). For independent and concurrent analysis, the scientists further combined multichannel microfluidics with the nanohole sensor arrays. Several capture antibodies were used to functionalise the sensing surface in order to confer molecular selectivity [191]. The optical transmission spectrum peaks would redly shift as a result of a change in the local refractive index upon specific binding of EVs. Quantitative analysis of EV proteins was made possible by the correlation between the amplitude of the spectral shift and the molecular mass density covering the sensor surface [189]. Using a sequence of titration experiments, our label-free nPLEX test showed an improved detection limit of around 3000. Quantitative analysis of EV proteins was made possible by the correlation between the amplitude of the spectral shift and the molecular mass density covering the sensor surface. This label-free nPLEX test showed a better limit of detection of around 3000 vesicles (670 aM) by the use of a series of titration experiments. This is 10^2^ and 10^4^ more sensitive than Western blotting and chemiluminescence ELISA, respectively. Importantly, the nPLEX demonstrated outstanding accuracy across a variety of protein indicators when compared to gold standard ELISA readings. With little processed materials, the full assay might be completed in less than 30 min, which makes the system appealing for quick clinical applications [189].

Currently, this technology is being developed for commercial use. Sensitive label-free biomolecular binding event detection at a metal-dielectric interface may be achieved by surface plasmon resonance (SPR). Particularly, when a surface plasmon is contained in nanoparticles that are the same size as the incident light wavelength or smaller, localised surface plasmon resonance (LSPR) takes place [[191], [192], [193]]. As opposed to oscillating over the metal-dielectric contact, the induced plasmons in LSPR fluctuate locally to the nanostructure. Consequently, with LSPR, the electromagnetic field decays far more quickly. This reduced field decay duration (100 nm) increases sensitivity to minute biomolecular interaction at the surface while mitigating sensor interference from bulk refractive index variations. Joshi et al. recently created a sensitive miRNA sensor by attaching chemically produced gold nanoprisms to a solid substrate, capitalizing on the benefit of LSPR for the detection of tiny biomolecules [194]. Two procedures were used to build the sensor: (1) chemical synthesis of ∼40 nm edge-length gold nanoprisms and their covalent attachment to a glass substrate; and (2) functionalisation of the immobilized nanoprisms with capturing DNA probes and polyethylene glycol spacers [194]. The LSPR resonant peak changed when the miRNA target (miR-10b) hybridised with the capturing DNA probes. The sensor achieved great detection sensitivity, with a limit of detection of 91 aM, because to its sharp nanoprism tips that strongly enhanced the electromagnetic field, and its atomically flat surface that allowed for homogenous packing.

## 7 Pre-analytical considerations

Sample collection for exosome isolation must be meticulously planned and executed to ensure the integrity and reliability of the results. The type of fluid being collected, such as blood, urine, or saliva [195], dictates the specific protocols and types of collection containers used, often incorporating additives like EDTA for blood to prevent clotting. It's crucial to collect sufficient volumes to ensure adequate exosome yield while considering practical and ethical limits, especially in clinical settings. During collection, steps must be taken to avoid contamination, ensuring sterile techniques are employed to prevent microbial intrusion and minimize cellular debris, which could compromise the sample. Additionally, it is generally recommended to process samples swiftly after collection to prevent the degradation of exosomes; if immediate processing isn't possible, the samples should be promptly stabilized and stored under appropriate conditions to preserve their viability for later analysis [196]. Proper storage is crucial for preserving the quality of exosome-containing samples and involves several key considerations. Typically, samples should be frozen at −80 °C to maintain the structural and functional integrity of the exosomes, and it's essential to avoid repeated freeze-thaw cycles, which can damage the exosomes and lead to the degradation of their contents [197,198]. The duration of storage also needs careful attention; although exosomes can be stable for several months under optimal conditions, the specific storage time should be validated to ensure that there is no significant deterioration in exosome quality [[198], [199], [200]]. Handling practices are equally important—samples should always be kept on ice during preparation and handling, and exposure to room temperature should be minimized. In some cases, the addition of stabilizers or the use of specialized storage containers can further enhance the stability and quality of exosomes during long-term storage, ensuring that they remain suitable for future analyses [[201], [202], [203], [204]].

## 8 Challenges in exosome isolation

Isolating exosomes from various biological fluids presents a series of challenges that significantly impact the purity, yield, and overall quality of the samples obtained, crucial factors for both research and clinical applications. One of the foremost difficulties in exosome isolation is the dual challenge of achieving high purity while maintaining a satisfactory yield. Many of the conventional isolation techniques, such as ultracentrifugation and polymeric precipitation, often force a trade-off between these two factors. Ultracentrifugation, for example, is favored for its ability to yield highly pure exosomes, but it often does so at the cost of lower overall yield [205,206]. Conversely, methods like polymeric precipitation can provide a higher yield but typically include a higher contamination rate from other extracellular vesicles and proteins.

Compounding the issue, exosomes inherently share similar size ranges and biophysical properties with other types of extracellular vesicles, such as microvesicles and apoptotic bodies. This similarity makes the selective isolation of exosomes particularly challenging without the use of specific markers that can accurately distinguish between these vesicle types. Additionally, the variability in exosome concentration and volume available from different sources (e.g., blood, urine, cerebrospinal fluid) necessitates adaptable isolation approaches, complicating the standardization of isolation protocols across different labs and studies. The equipment required for some of the more precise isolation methods, such as advanced ultracentrifugation setups and microfluidic devices, represents a significant investment and is not universally available in all research settings. This discrepancy in equipment availability can lead to variability in the quality and reproducibility of exosome isolations performed under different conditions or in different laboratories.

Moreover, the field suffers from a notable lack of standardized protocols, which exacerbates difficulties in comparing and replicating results across different studies. This lack of standardization stems from the diversity in the types of body fluids used, the variable nature of exosomes themselves, and the assortment of isolation techniques employed. Pre-analytical factors such as the choice of anticoagulants, the time elapsed between sample collection and processing, and the conditions under which samples are stored can all influence the integrity and yield of the isolated exosomes. Establishing standardized conditions for these factors is critical for the development of reliable, reproducible isolation protocols [207,208].

Finally, establishing effective quality control measures for isolated exosomes is another significant challenge, given their heterogeneity in size, composition, and cellular origin [209,210]. This variability makes it difficult to define uniform quality benchmarks that can be universally applied to assess the quality of exosome preparations [211,212]. Developing robust, standardized assays to evaluate the purity, size distribution, and functional integrity of exosomes is essential for advancing their use in clinical diagnostics and therapeutic applications, ensuring that exosome-based studies and treatments are both reliable and effective [[213], [214], [215], [216]].

## 9 Exosome heterogeneity, intercellular communications & therapeutic potential in cancer

**Fig. 14:**
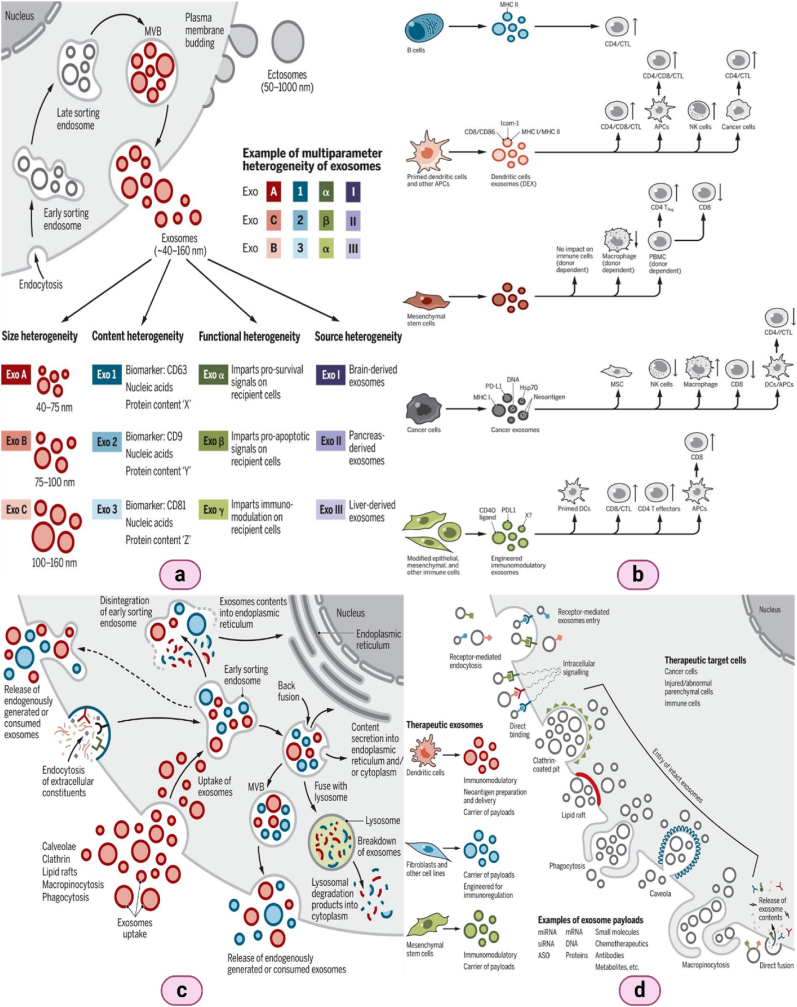
**(a)** The heterogenicity of extracellular vesicles and exosomes; **(b)** Exosome's role in modulating the immune response; **(c)** The intracellular pathways of exosomes that are internalized and those produced within the cell; **(d)** The process of cellular absorption of therapeutic exosomes. (Reproduced with Creative Commons CC BY 4.0 license, sourced from Ref. [169], Copyright © 2020 by the Authors).

Exosomes display a remarkable degree of heterogeneity, reflecting the diverse conditions and cellular contexts from which they originate. This variability is evident in their size, molecular composition, and functional capacities. The molecular makeup of exosomes is not arbitrary but is intricately linked to the physiological and pathological state of the parent cell [217,218]. For instance, exosomes derived from tumor cells differ significantly from those released by normal cells in terms of their cargo and membrane composition. This heterogeneity is primarily driven by the cells’ differential use of the multiple pathways involved in exosome formation, such as ESCRT-dependent and independent mechanisms, as well as the specific cargo sorting based on post-translational modifications and lipid content [[219], [220], [221]] (Fig. 14).

The diversity in exosome composition is crucial for their functional specialization, enabling them to selectively interact with specific recipient cells and modulate a variety of biological processes [222,223]. This selective interaction is mediated by specific surface proteins and lipids that dictate the targeting and fusion of exosomes with recipient cell membranes, influencing cell behaviour in a highly controlled manner [224,225]. The heterogeneity of exosomes thus plays a critical role in the specificity and efficacy of intercellular communication, allowing for tailored responses to various microenvironmental cues and cellular needs.

Exosomes are central players in intercellular communication, capable of transferring a wide array of bioactive molecules between cells over both short and long distances [226,227]. This communication is facilitated by the encapsulation of proteins, lipids, mRNAs, and microRNAs within exosomes, which can then be delivered to recipient cells [228,229]. Upon fusion or interaction with these cells, exosome cargoes can modulate recipient cell gene expression, signaling pathways, and overall behaviour [230].

The impact of exosomal communication is vast, influencing processes such as immune regulation, where exosomes can promote or suppress immune responses depending on their origin and cargo [231,232]. In the tumor microenvironment, cancer-derived exosomes contribute to tumorigenesis by promoting angiogenesis, suppressing the immune response, and enhancing metastatic behaviour through the transfer of oncogenic factors and signalling molecules [233,234]. Furthermore, exosomes can cross biological barriers, such as the blood-brain barrier, making them effective conveyors of information and functional molecules across otherwise impermeable cellular boundaries, thereby orchestrating complex multi-organ processes and responses.

The therapeutic potential of exosomes in cancer is vast and multifaceted, primarily revolving around their capabilities for targeted drug delivery and as vehicles for immunotherapy. Engineered exosomes can be loaded with chemotherapeutic agents, small interfering RNAs (siRNAs), or CRISPR-Cas9 gene editing systems, and directed to specific cancer cells, thus minimizing off-target effects and enhancing therapeutic efficacy [235,236]. This targeted delivery is particularly advantageous due to the natural homing abilities of exosomes, which can be exploited to deliver therapeutic payloads directly to tumor sites or metastatic niches.

Additionally, exosomes are being explored in the realm of cancer immunotherapy. Exosomes derived from dendritic cells, for instance, can be loaded with tumor antigens and used to stimulate an immune response against cancer cells. The presence of major histocompatibility complex (MHC) molecules on exosomes allows them to effectively present antigens to T cells, triggering an immune response that can lead to the destruction of tumor cells.

Moreover, the use of exosomes as biomarkers in cancer diagnostics offers significant clinical value. Due to their stability in bodily fluids and the specific molecular signatures they carry from their cells of origin, exosomes can be used for non-invasive monitoring of tumor progression, response to treatment, and detection of recurrence, thereby significantly improving the management and prognosis of cancer patients. The exploration of exosome heterogeneity, their role in intercellular communication, and their therapeutic potential in cancer opens up new avenues for understanding and treating this complex disease. Harnessing the unique properties of exosomes could revolutionize cancer therapy, turning exosome research into a cornerstone of future oncological interventions.

## 10 Future perspectives

Advances in exosome isolation techniques have significantly improved the efficiency, specificity, and practicality of exosome research and clinical diagnostics. These innovations address some of the limitations of traditional methods, enhancing both the quality and the utility of isolated exosomes. Microfluidic devices have been refined to allow precise manipulation of fluids at microscopic scales, facilitating the isolation of exosomes with high purity and efficiency. These devices integrate multiple functions, such as capture, washing, and elution, into a single platform. They can be designed to target exosomes based on size, density, or specific surface markers, and are particularly useful for isolating specific exosome subpopulations from small sample volumes. Recent developments in immunoaffinity-based techniques have included the use of magnetic nanoparticles coated with antibodies specific to exosomal proteins. This approach enhances the selectivity and purity of isolated exosomes by targeting distinct biomarkers and allows for the easy separation of exosomes using a magnetic field, reducing sample handling and processing time.

Innovations in ultracentrifugation include the use of density gradient media, which improves the purity of exosomes by minimizing the co-isolation of other vesicles and particles. Modified ultracentrifugation protocols have also been developed to reduce processing times and improve yield. New formulations of precipitation agents have been designed to increase the specificity and efficiency of polymeric precipitation. These advancements aim to reduce the co-precipitation of non-exosomal proteins and improve the overall purity of the isolated exosomes. Also, two advanced technologies are utilized nowadays; Automated and High-throughput Systems and nanotechnology-based Approaches. Automated and high-throughput systems for exosome isolation streamline the process by integrating multiple steps into a single, cohesive workflow, enhancing efficiency, consistency, and scalability. The procedure begins with system setup, including calibration and protocol loading, followed by automated sample loading where biological fluids are prepared and processed to remove cells and debris. The system then handles the addition of reagents, such as binding buffers or precipitation solutions, and manages incubation and separation steps, such as magnetic bead-based isolation, with precise control over timing and conditions. After capturing the exosomes, automated washing steps remove unbound materials, and the exosomes are eluted using specific buffers or changes in physical conditions. The isolated exosomes are collected in designated containers, with all data recorded for traceability and quality control. The system concludes with an automated cleaning cycle to prevent cross-contamination and maintenance checks to ensure optimal operation. This automation not only improves reproducibility and reduces human error but also supports high-throughput processing, essential for large-scale research and clinical diagnostics.

In research, automated high-throughput systems allow scientists to conduct large-scale studies of exosomes with a level of precision and reproducibility that manual methods cannot match. This capability is crucial for biomarker discovery, where the identification and validation of new markers require the analysis of large datasets to establish statistical significance.

In clinical settings, the consistency and reliability of automated systems support the use of exosomes as diagnostic and prognostic tools. In cancer diagnostics, exosomes can be isolated from patient blood samples and analyzed for specific biomarkers that indicate the presence or progression of a tumor. Automated systems ensure that this process is fast, reliable, and compatible with the workflow of clinical laboratories.

Nanotechnology-based approaches for exosome isolation leverage the precision of nanoscale materials to enhance capture efficiency and specificity. Initially, nanoparticles such as gold or magnetic nanoparticles are synthesized and then functionalized with molecules that target specific exosomal surface proteins, like antibodies against CD63, CD9, or CD81. Biological samples, such as blood or urine, are pre-processed to remove cells and debris, and then incubated with these functionalized nanoparticles to allow exosomes to bind specifically to them. After binding, the nanoparticle-exosome complexes are isolated from the sample using methods appropriate to the nanoparticle type, such as magnetic fields for magnetic nanoparticles. These complexes are then washed to remove non-specifically bound materials, and exosomes are eluted using changes in buffer conditions or competitive binding. The isolated exosomes can then be analyzed using techniques like nanoparticle tracking analysis or electron microscopy to assess their purity, quantity, and integrity. While this method offers high specificity and can be integrated into complex diagnostic tools, it requires careful handling and optimization to maintain nanoparticle stability and functionality, posing challenges in scalability and cost for broader clinical applications.

## 11 Exploring the frontier of exosome research in cancer clinical trials

**Fig. 15:**
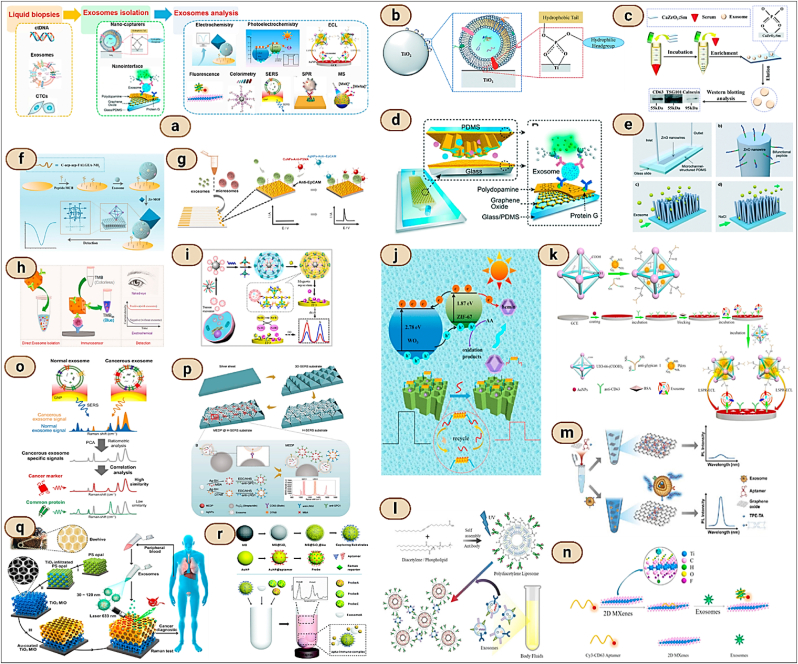
**Nanomaterials assisted exosomes isolation.** (Reproduced with permission under Creative Commons CC BY 4.0 license from Ref. [238] Copyright @ 2022 The Authors).

Clinical trials centered on exosomes are spearheading advancements in oncology, exploring their roles as both markers for early detection and avenues for targeted therapy. These microscopic extracellular vesicles are packed with diverse biomolecules like proteins, DNA, RNA, and lipids, which play significant roles in the dynamics of cancer progression and the spread of metastatic cells. Within clinical environments, researchers are capitalizing on the diagnostic and prognostic properties of exosomes by studying their molecular profiles and interactions within patients. Some trials strategically focus on extracting exosomes from fluids such as blood and urine to identify early signs of cancer or to track the effectiveness of treatments. Furthermore, investigations into tumor-derived exosomes examine their ability to either enhance or suppress tumor growth, which may lead to innovative treatments that interrupt these critical signaling pathways. There is also growing interest in utilizing exosomes for direct drug delivery to tumor sites, which promises to increase treatment efficacy while reducing systemic side effects. The breakthroughs from these trials are expected to revolutionize the approach to cancer treatment, enabling more personalized and effective strategies [237] ( Fig. 15).

**Fig. 16:**
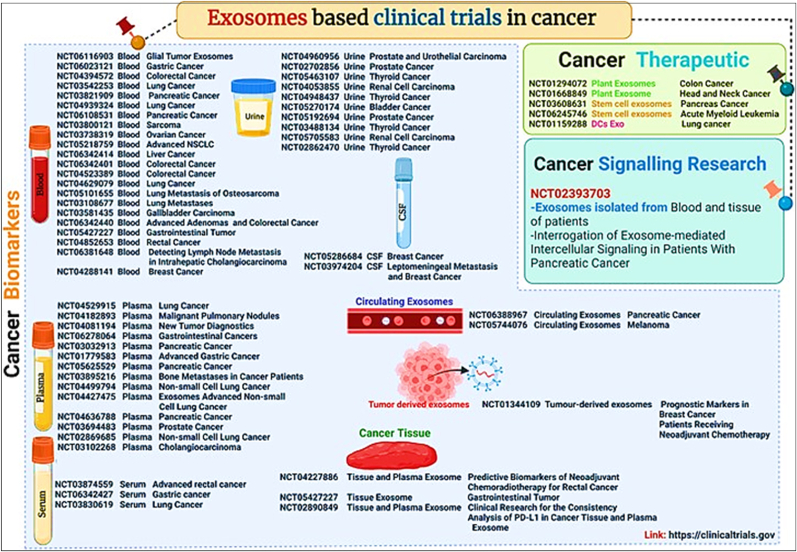
Exosomes-based clinical trials of exosomes. (Reproduced with permission under Creative Commons CC BY 4.0 license from Ref. [237] Copyright @ 2022 The Authors).

Fig. 16 offers an insightful aggregation of various clinical trials that probe the role of exosomes in oncology, highlighting the diverse types of cancer addressed and specific operational areas these studies target. Organized into categories such as Cancer Biomarkers, Circulating Exosomes, Cancer Tissue, and focused research on Therapeutics and Signaling, each study is listed with its NCT number, a unique code registered on ClinicalTrials.gov that provides detailed information about each trial. These studies encompass a wide range of cancers, including lung, pancreatic, colorectal, and breast cancers, focusing on utilizing exosomes for diagnostics, prognostics, therapeutic interventions, and exploring cancer signaling pathways. This structured overview underscores the vital role of exosomes in enhancing cancer diagnostics and therapy, showcasing their potential to facilitate a shift towards personalized medical approaches through intricate analysis of exosomal content and activity in varied biological contexts.

## 12 Summary and future perspectives

The evolving field of exosome research and diagnostics, effective isolation techniques are crucial for advancing our understanding and application of these extracellular vesicles [239,240]. Exosomes, due to their role in intercellular communication and disease processes, have significant potential as biomarkers and therapeutic agents [241,242]. Traditional isolation methods like ultracentrifugation, while established, face challenges in terms of time consumption, equipment requirements, and balancing purity with yield. Newer techniques, such as size-based isolation and immunoaffinity capture, offer improvements but come with their own set of limitations, such as potential sample contamination and high costs.

**Fig. 17:**
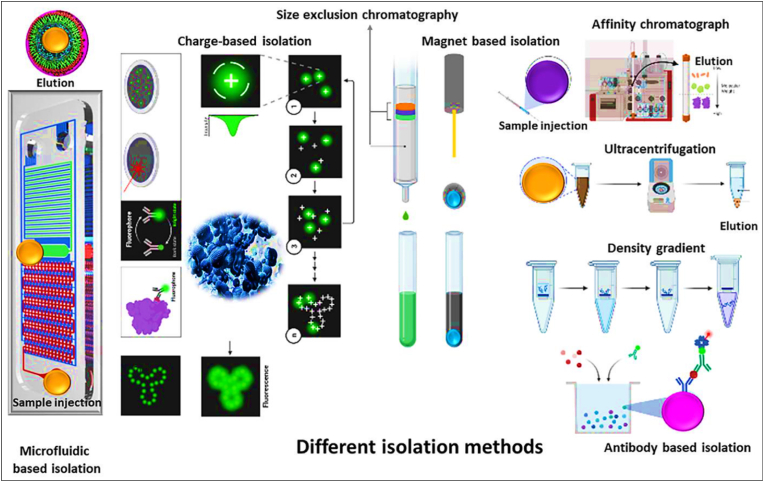
**Representation of EVs isolation and purification by various methods.** (Reproduced with permission under Creative Commons CC By 4.0 license from Ref. [243] Copyright @ 2022 The Authors).

Nanotechnology-based approaches bring innovative solutions by using nanoscale materials to enhance the specificity and efficiency of exosome capture, while automated and high-throughput systems streamline the isolation process, ensuring consistency and scalability [[243], [244], ]. These advanced systems reduce human error, improve reproducibility, and allow for the processing of large sample volumes, making them particularly valuable in clinical diagnostics and large-scale research. Despite these advances, challenges remain in standardizing protocols across different methods and ensuring the biocompatibility and stability of materials used in isolation processes (Fig. 17).

The field stands at a pivotal juncture, with evolving isolation techniques significantly enhancing our ability to harness the diagnostic and therapeutic potential of exosomes. Advances in isolation techniques have not only increased the specificity and efficiency of exosome isolation but also paved the way for innovations that could transform medical diagnostics and treatment modalities. To overcome the limitations, the field must push for the development of global standards in exosome isolation and characterization. This could include defining key performance indicators for isolation techniques and establishing benchmark protocols that ensure consistency across studies. Furthermore, fostering collaborative research initiatives and sharing best practices can accelerate the standardization process. There is also a pressing need to innovate cost-effective and user-friendly technologies that democratize access to advanced methodologies, enabling wider adoption in clinical settings. Automating isolation processes and integrating artificial intelligence could streamline workflows, enhance reproducibility, and reduce operational costs. By addressing these challenges head-on and leveraging collaborative and technological innovations, the research community can unlock the full potential of exosomes in revolutionizing disease diagnosis, monitoring, and treatment, ushering in a new era of targeted and personalized healthcare solutions.

The continued refinement of exosome isolation techniques, including the adoption of nanotechnology and automation, is essential for realizing the full potential of exosomes in diagnostics and therapeutics. As these technologies advance, they promise to provide more reliable, efficient, and scalable solutions, paving the way for innovative applications in personalized medicine and beyond.

## CRediT authorship contribution statement

**Nobendu Mukerjee:** Writing – review & editing, Writing – original draft, Visualization, Validation, Software, Resources, Project administration, Methodology, Investigation, Formal analysis, Data curation, Conceptualization. **Arghya Bhattacharya:** Writing – review & editing, Writing – original draft, Visualization, Validation, Software, Resources, Methodology, Investigation, Formal analysis, Data curation, Conceptualization. **Swastika Maitra:** Writing – review & editing, Writing – original draft, Visualization, Validation, Software, Resources, Methodology, Investigation, Formal analysis, Data curation, Conceptualization. **Mandeep Kaur:** Writing – review & editing, Writing – original draft, Visualization, Validation, Software, Project administration, Methodology, Investigation, Formal analysis, Data curation, Conceptualization. **Subbulakshmi Ganesan:** Writing – review & editing, Writing – original draft, Visualization, Validation, Software, Methodology, Investigation, Formal analysis, Data curation, Conceptualization. **Shivang Mishra:** Writing – review & editing, Writing – original draft, Visualization, Validation, Software, Methodology, Investigation, Formal analysis, Data curation, Conceptualization. **Ayash Ashraf:** Writing – review & editing, Writing – original draft, Visualization, Validation, Software, Methodology, Investigation, Formal analysis, Data curation, Conceptualization. **Muhammad Rizwan:** Writing – review & editing, Validation, Investigation, Formal analysis. **Kavindra Kumar Kesari:** Writing – review & editing, Writing – original draft, Visualization, Validation, Supervision, Software, Resources, Project administration, Methodology, Investigation, Funding acquisition, Formal analysis, Data curation, Conceptualization. **Tanveer A. Tabish:** Writing – review & editing, Visualization, Validation, Supervision, Resources, Project administration, Methodology, Investigation. **Nanasaheb D. Thorat:** Writing – review & editing, Writing – original draft, Visualization, Validation, Supervision, Software, Resources, Project administration, Methodology, Investigation, Funding acquisition, Formal analysis, Data curation, Conceptualization.

## Declaration of competing interest

The authors declare that they have no known competing financial interests or personal relationships that could have appeared to influence the work reported in this paper.

## Data Availability

No data was used for the research described in the article.

## Abbreviations:

EV: Extracellular Vesicles
MVB: Multivesicular Bodies
ILVs: Intraluminal Vesicles
ESCRT: Endosomal Sorting Complex Required for Transport
miRNA: microRNA
mRNA: messenger RNA
PEG: Polyethylene Glycol
SEC: Size-Exclusion Chromatography
CSF: Cerebrospinal Fluid
MHC: Major Histocompatibility Complex
siRNAs: Small Interfering RNAs
CRISPR-Cas9: Clustered Regularly Interspaced Short Palindromic Repeats and CRISPR-associated protein 9
CD63, CD9, CD81: Cluster of Differentiation 63, 9, 81 (exosome markers)
RNA: Ribonucleic Acid
DNA: Deoxyribonucleic Acid
